# The Roles of Fluid Intelligence and Emotional Intelligence in Affective Decision-Making During the Transition to Early Adolescence

**DOI:** 10.3389/fpsyg.2020.574903

**Published:** 2020-12-16

**Authors:** Danfeng Li, Mengli Wu, Xingli Zhang, Mingyi Wang, Jiannong Shi

**Affiliations:** ^1^School of Sociology and Psychology, Central University of Finance and Economics, Beijing, China; ^2^CAS Key Laboratory of Behavioral Science, Institute of Psychology, Chinese Academy of Sciences, Beijing, China; ^3^Department of Psychology, University of Chinese Academy of Sciences, Beijing, China; ^4^Department of Psychology, School of Humanities and Social Sciences, Beijing Forestry University, Beijing, China; ^5^Department of Learning and Philosophy, Aalborg University, Aalborg, Denmark

**Keywords:** affective decision-making, Iowa gambling task, fluid intelligence, emotional intelligence, early adolescence

## Abstract

The current study mainly explored the influence of fluid intelligence (IQ) and emotional intelligence (EI) on affective decision-making from a developmental perspective, specifically, during the transition from childhood into early adolescence. Meanwhile, their age-related differences in affective decision-making were explored. A total of 198 participants aged 8–12 completed the Iowa Gambling Task (IGT), the Cattell’s Culture Fair Intelligence Test and the Trait Emotional Intelligence Questionnaire-Child Form. Based on the net scores of IGT, the development of affective decision-making ability did not increase monotonically with age, and there was a developmental trend of an impaired IGT performance in early adolescence (aged 11–12), especially in the early learning phase (first 40 trials) of the IGT. More importantly, IQ and EI played different roles for children and early adolescents: IQ and EI jointly predicted the IGT performance for 8–10 years old children, whereas only EI contributed to the IGT performance of 11–12 years old early adolescents. The present study extends the evidence how cognitive processing and emotional processing interact in affective decision-making from the developmental perspective. Furthermore, it provides insights of future research and intervention with early adolescents’ poor affective decision-making.

## Introduction

Adolescence is a period of increasing emotional sensitivity and poor self-control that is often accompanied by risky behaviors and poor decision-making, such as drug abuse, binge drinking, reckless driving, unprotected sex, or criminal activities (Arnett, 1992; Casey et al., 2008; Steinberg, 2008; Prencipe et al., 2011). Furthermore, Defoe et al. (2015) found that early adolescents (begins at the age of 11–12) took more risks than mid-late adolescents (aged 14–19). Ill-consider decisions made during early adolescence might carry life-long consequences thereafter, which includes poverty, poor well-being, and even mortality (Spear, 2000; Mahalik et al., 2013). Their maturational imbalance between a slowly developed “cold” system (cognitive) and the excessive “hot” system (emotional) leads to their impaired decision-making ability in early adolescence (Blakemore, 2008; Romer, 2010).

Decision-making about future events with emotionally significant consequences such as potential rewards and punishments has been termed affective decision-making (Kerr and Zelazo, 2004; Torralva et al., 2007; Xiao et al., 2009). Affective decision-making is a typical “hot” executive function, and it is related to the prolonged development of prefrontal cortex, PFC, which continues to develop into adolescence, even early adulthood (Zelazo et al., 2008). Iowa Gambling Task (IGT) is the most widely used paradigm for evaluating individual affective decision-making including children, adolescents and adults in that it involves unpredictable gains or losses similar to real-life choices (Bechara et al., 1994). Participants are expected to maximize their gains by choosing cards from four decks without knowing what the cards would yield in advance. Two decks of the cards are advantageous decks with less immediate rewards but overall net gains, whereas, the other two appear to be appealing at first by bringing more immediate payoffs but overall net losses. Thus, the core feature of IGT is to forgo short-term interest for benefits in the long run (Dunn et al., 2006). The total net scores are used to assess the performance in IGT, which can be calculated by subtracting the total number of disadvantageous decks from total advantageous decks. In addition, net scores in each block (every 20 trials as a block) can be analyzed to see how affective decision-making changed over the course of the IGT (Bechara et al., 1997). A higher and positive difference score indicates a more successful performance in affective decision-making.

To date, the developmental trajectory of children and adolescents’ decision-making ability as measured by IGT performance is still unclear. On the one hand, some studies found that advantageous decision-making in IGT has improved with age. For example, Prencipe et al. (2011) divided children and adolescents into four groups: 8–9, 10–11, 12–13, and 14–15 years. Their results showed a linear pattern of the development of affective decision-making. Moreover, Crone and van der Molen (2004) used an adapted version of IGT and found that adults (18–25 years) performed better than adolescents (13–15 years), and adolescents (13–15 years) performed better than children and preadolescents (6–9 and 10–12 years). Meanwhile, Hooper et al. (2004) grouped participants (aged 9–17) into three age bands and found that 14–17 years old group made more advantageous IGT choices compared with the other two younger groups. These findings all indicated that the development of affective decision-making continues to adolescence and even adulthood, which is due to the protracted development of the prefrontal cortex (Zelazo et al., 2008). On the other hand, another significant study (Smith et al., 2012) proposed a J-curve model (quadratic function curve) of development, showing an impairment of IGT performance in early adolescents (around age 12) as well as a linear improvement from age 14 and a peak at age 17. Smith et al. (2012) have used the “neurodevelopmental imbalance models” to explain deficient decision-making of early adolescents: the maturational imbalance of the overactive socioemotional system (high reward sensitivity) and the slow-developed cognitive control system (Blakemore, 2008; Romer, 2010; Quinn and Harden, 2013).

As we noticed, the main inconsistency among previous findings concerning the development of affective decision-making in children and adolescents appears only during early adolescence (aged 11–12). We believe that the mixed findings could be the result of methodological differences. Specifically, compared with wider age-range measurements as Crone and van der Molen (2004); Hooper et al. (2004), and Prencipe et al. (2011) did, a continuous age selection of participants used in the study of Smith et al. (2012) enables a detailed exploration of subtle differences during the transition from late childhood into early adolescence with regard to affective decision-making ability. However, in the study of Smith et al. (2012), the inadequate number of children and adolescent samples of each group (unevenly ranged from 8 to 18) may make their final results less robust. Therefore, we not only adopt continuous sampling by setting five age groups from 8 to 12, but increase the sample size of each group to over 35, which may help us further explore whether there is an early adolescent-specific (aged 11–12) decrease in the development of affective decision-making in the current study.

Many researchers have pointed out that cognition and emotion are the two major drivers that could influence children and adolescents’ IGT performance. For example, some researchers found that memory and learning could account for age-related improvements in advantageous decision making in IGT (Van Duijvenvoorde et al., 2012), whereas other researchers proposed that rewards and/or losses in IGT were tied to a relatively strong emotional component (Carlson et al., 2009; Prencipe et al., 2011). In addition, the dual systems model has often been used to explain the developmental mechanism in children and adolescents’ decision-making. The neurodevelopmental imbalance model (one of dual systems model) is divided into socioemotional system (“hot” system, emotional) and the cognitive control system (“cold” system, cognitive) (Galvan et al., 2006; Pfeifer and Peake, 2012; McClure and Bickel, 2014). The former system (connecting the ventral tegmental area and the nucleus accumbens, NAc) responds to pleasant, novel, and rewarding stimuli, and it reaches its apex during early adolescence (Van Leijenhorst et al., 2010; Christakou et al., 2011; Shulman et al., 2016). However, the latter system (governed by PFC) refrains unwise impulses, and it continues to mature into early adulthood (Veroude et al., 2013; Bezdjian et al., 2014).

Cattell-Horn-Carroll theory (CHC) of cognitive abilities has been regarded as a common taxonomy for intelligence researchers (McGrew, 2009), and in this taxonomy, cognitive abilities are placed on the three strata: III (g-general), II (broad abilities, such as fluid intelligence and crystallized intelligence), I (narrow abilities, such as inductive reasoning) (McGrew, 2009). In laboratory research, existing studies have found that fluid intelligence (IQ) could predict IGT performance for both healthy and clinical adult samples (Johnson et al., 2006; Fein et al., 2007; Demaree et al., 2010; Webb et al., 2014). Compared with adult studies, only a few studies tapped the relationship between IQ and IGT of children and early adolescents. Most of the existing research has not found that IQ could predict children’s and early adolescents’ affective decision-making ability (Lehto and Elorinne, 2003; Crone and van der Molen, 2004). However, recent research indicates different findings. For example, gifted children outperformed their average peers in regards of decision-making strategies and speed, which suggests that IQ can predict the affective decision-making ability of children and early adolescents (Li et al., 2017). Another study (Flouri et al., 2019) with over 12,000 participants found that intelligence was substantively associated with the risk adjustment and quality of decision-making measured by the performance of Cambridge Gambling Task in early adolescence (aged 11). By comparing the different findings of existing papers in IGT, we believe that the inconsistent results are due to different choice of indices. Specially, Crone and van der Molen (2004) only used the overall indicator (i.e., the number of selected advantageous cards) to measure IGT performance of children and adolescents, whereas Li et al. (2017) used a variety of indicators, including the overall indicators of IGT performance (i.e., total net scores) and indicators of the decision process (the net scores in five block). Therefore, in the present study, we use multiple indices of IGT performance including the overall net score and the net score of each block.

The somatic marker hypothesis (SMH) supports the idea that emotional processes play an essential role in the IGT. The SMH postulates that people acquired emotion-based biasing signals generated from the somatic markers (i.e., the internal environment, viscera, bones, and smooth muscles), which enable individuals to make wiser choices in IGT (Damasio, 1996). Prior studies found that patients with damage in ventromedial prefrontal cortex (VMPFC) had poorer performance in IGT when compared with the control group, despite their intact IQ (Damasio, 1994). Apart from the VMPFC, the somatic maker circuity (SMC) also includes the amygdala, insula, anterior cingulate, somatosensory cortex, and basal ganglia (Bechara and Damasio, 2005; Dunn et al., 2006). Furthermore, Bar-On et al. (2003) has proposed one model that emotional intelligence (EI) abilities rely heavily on the SMC. EI is defined as accurately assessing the emotions of oneself and others, properly expressing emotions, and the ability to adaptively regulate emotions (Mayer and Salovey, 1993). Many experimental studies have also explored the relationship between EI and IGT performance. On the one hand, some studies have found that EI positively predict IGT performance, that is, individuals with higher EI, were better at using emotional cues in affective decision-making and thus perform better (Sevdalis et al., 2007; Telle et al., 2011). Moreover, a recent study showed that training of EI could lead to improved IGT performance for healthy adults (Alkozei et al., 2019). On the other hand, EI-IGT relationship was not observed in other studies (Demaree et al., 2010). We believe that two reasons might account for the inconsistency. Firstly, different measurements of EI could produce different results. The Schutte Emotional Intelligence Scale (SEI) used by Demaree et al. (2010) is based on the ability EI model, whereas the Trait Emotional Intelligence Questionnaire (TEIQue) used by Sevdalis et al. (2007) and Telle et al. (2011) is based on trait EI model. Ability EI refers to the ability to perceive, express, understand and regulate emotion in the self and others (Mayer and Salovey, 1993), and it reflects more of cognitive ability (Petrides, 2011). Trait EI focuses on individual emotional self-perceptions (Petrides and Furnham, 2001) and thus it is believed to be a better EI model to explore how emotion affects affective decision-making from the individual perspective when compared with ability EI (Sevdalis et al., 2007). Secondly, many studies only stayed on the surface of total score of EI and rarely explore the sub-dimensions of EI, thus leaving some significant findings uncovered. For example, researchers found that emotion awareness as a facet of EI negatively predicted the advantageous card choices in the IGT for female students (Pilárik and Sarmany-Schuller, 2009). Therefore, in the present study, we will use trait EI measurement to assess EI and its total nine facets according to Mavroveli et al. (2008). It includes adaptability, emotion perception, emotion expression, self-motivation, self-esteem, low impulsivity, peer relations, emotion regulation, and affective disposition. In this way, we can further analyze the relationship between EI and IGT, and might shed light for pertinent future training in children and adolescents.

Up to the present time, rarely do studies simultaneously examine the predictive effects of IQ and EI on the IGT during the transition from childhood into early adolescence. Among the existing papers with adult samples, Demaree et al. (2010) and Webb et al. (2014) both indicated that adults’ IGT performance was more dependent on IQ (cognitive processing) compared to EI (emotional processing). In addition, Li et al. (2017) found that the performance of IGT for 9 years old children was jointly predicted by IQ and EI. Meanwhile, another recent longitudinal study across 10 years span found that cognitive and affective variables were substantively associated with decision-making ability in early adolescence (Almy et al., 2018). These findings showed that IQ and EI may simultaneously predict children and early adolescents’ affective decision-making, but this assumption also deserves more exploration to confirm it. Based on the developmental literature and the dual systems model, young children’s socioemotional system and the cognitive control system are both in earlier phase of maturation (Rueda et al., 2004). Therefore, their IGT performance might be associated with both EI and IQ. Conversely, for early adolescents, due to the hyperactivity of the NAc driven by rewards and the underdeveloped PFC control (Figner and Weber, 2011), their socioemotional system is very likely to prevail over the cognitive control system. Hence, EI and IQ might both matter but EI could be more related to affective decision-making for early adolescents. In addition, early adolescents are more likely to perform less advantageously because they are more sensitive to high reward decks compared to their younger counterparts.

The aim of present study is to examine the age-related differences in affective decision-making as measured by IGT performance during the transition from childhood into early adolescence (8–12 years old), especially with a focus on whether early adolescents (11–12 years old) have deficits in affective decision-making. Moreover, this research is to explore the relationship among IQ, EI, and affective decision-making ability for children and early adolescents. It is beneficial for us to understand how cognitive processing and emotional processing interact in affective decision-making from a developmental perspective, and to tailor the training and intervention for early adolescents with poor affective decision-making ability. We hypothesized that (1) the IGT performance of children and early adolescents will progress in a non-monotonic way. Specifically, early adolescents (aged 11–12) may demonstrate a deficit compared to young children (aged 8–10), and (2) for children, IQ and EI contribute equally to IGT performance, and (3) for early adolescents, IQ and EI might jointly predict the IGT performance but EI would play a more significant role.

## Materials and Methods

### Participants

G^∗^Power 3.1 (Faul et al., 2007) was used to compute required sample size by a prior power analysis. According to previous research, we set 1 – β = 0.80, *α* = 0.05 and effect size *f* = 0.25. Two hundred participants were required for one-way ANOVA and two repeated measures ANOVAs. One hundred and ninety-eight children and early adolescents aged 8–12 years old were recruited from one local public primary school in the current study. Six participants did not complete the IGT seriously, and five others failed to complete the IGT due to computer errors, leaving the final sample of 187 participants. In the present study, we not only recruited relative sufficient number of participants, but also guaranteed there were more than 35 samples in each age group (see details in Table 1).

**TABLE 1:** Information of participants: age, gender, cognitive intelligence and emotional intelligence.

		Gender		Age (years old)		IQ		EI	
Age group (years old)	*N*	Male	Female	*M*	*SD*	*M*	*SD*	*M*	*SD*
8	39	20	19	8.12	0.29	22.41	6.84	3.83	0.44
9	38	21	17	9.12	0.30	28.92	6.48	3.91	0.46
10	39	23	16	9.99	0.31	33.74	4.15	4.00	0.49
11	36	17	19	11.24	0.35	31.53	3.84	3.89	0.47
12	35	13	22	12.15	0.34	34.54	5.61	3.87	0.41

As part of this study, we tested the IQ and EI of participants with Cattell’s Culture Fair Intelligence Test and Trait Emotional Intelligence Questionnaire-Child Form (TEIQue-CF). The details of demographic characteristics and their IQ and EI scores of the participants are presented in Table 1. ANOVAs were used to analyze the IQ and EI related age differences in 8–12 years. Regarding the IQ total score, *F*(4, 182) = 29.71, *p* < 0.001, η^2^ = 0.40, 8 years old children had the lowest IQ total score, and 10 years old children and 12 years old early adolescents had the higher IQ total scores than 9 years old children, *p*s < 0.001. Regarding the EI total score, there was no significant difference among 8–12 years old children and adolescents, *F*(4, 182) = 0.73, *p* = 0.57, η^2^ = 0.02. The children’s and adolescents’ parents helped them sign the informed consent form and reported their children were free of clinical disorders and uncorrected visual impairment.

### Measures

#### IGT

**FIGURE 1:**
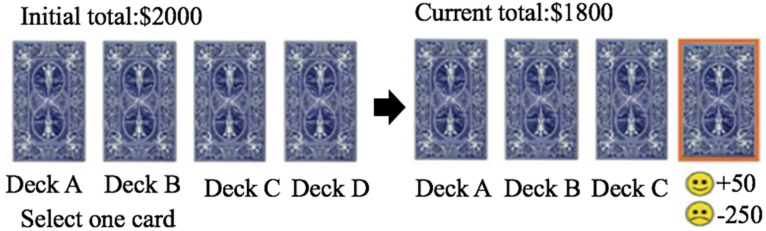
The flowchart of the IGT.

Participants completed a computerized version of the standard IGT programmed by E-prime 2.0 (Bechara et al., 1994). Initially, four decks of cards labeled A, B, C, and D appeared simultaneously on the computer screen, looking identical from the back. Participants started with a play amount of $2,000 and were asked to choose a card from the four decks on each trial in an effort to win as much money as possible. By pressing certain keys, corresponding decks would be chosen and the amount of rewards and punishments as well as the total points would appear on the screen (see details in Figure 1). Participants had to press the space bar to make their next choice until the end of IGT. All the participants received standard instructions without knowing how many trials they would have.

The gains and losses for each deck were set in advance. Deck A and Deck B yielded $100 for each time. However, in every 10 selections, Deck A brought five unpredictable losses of $150, $300, $200, $250, or $350 (high frequency), and Deck B brought an unpredictable loss of $1,250 (low frequency). In total, Deck A or Deck B revealed a net loss of $250, which made them disadvantageous decks. In contrast, Deck C and Deck D yielded $50 for each time. In every ten selections, Deck C brought five random losses of $25, $50, $75, $50, $50 (high frequency) while Deck D brought one random loss of $250 (low frequency). In total, Deck C or Deck D provided a net gain of $250, which made them advantageous decks.

In the present study, we employed the overall net score (100 trials) and net scores by block (every 20 trials as a block) to measure the IGT performance. The net scores were the difference between the number of advantageous decks (C + D) and the number of disadvantageous cards (A + B). As same as the standard IGT in Bechara et al. (1994), the IGT task in the present study had no practice block.

#### Fluid Intelligence

We used Cattell’s Culture Fair Test (Cattell and Cattell, 1973) to assess IQ in a paper-and-pencil form. Forty-six items were categorized into four subtests: sequence reasoning, homogenization generalization, square matrix reasoning, and qualitative analysis. Each item was assigned one point and the whole test would yield a total score for IQ (possible score range: 0–46). The CCFT exhibited adequate internal reliability of 0.74 (Cattell and Cattell, 1973), and the Cronbach’s alpha coefficient of this test was 0.72 in the present study.

#### Emotional Intelligence

We used the Trait Emotional Intelligence Questionnaire-Child Form (TEIQue-CF) to assess EI in a paper-and-pencil form. It was specifically designed for children and early adolescents aged 8–12 with comprehensive information concerning their personality facets of emotion (Mavroveli et al., 2008). The TEIQue-CF consists of 75 items, which were divided into nine facets: adaptability, affective disposition, emotion expression, emotion perception, emotion regulation, low impulsivity, peer relations, self-esteem, and self-motivation. Most of the items were statements describing the state of the emotions with responses on a 5-point Likert scale (i.e., “When I feel sad, I try to keep myself doing things”). The TEIQue-CF exhibited adequate internal reliability of 0.76 and a robust test–retest reliability of 0.79 (Mavroveli et al., 2008). The Cronbach’s alpha coefficient of TEIQue-CF was 0.84 in the present study.

### Procedure

All the participants completed the IQ and EI tests via simultaneous group testing in a fixed order. The IQ test was followed by the EI test. The total test lasted about 30–40 min. The IGT was conducted individually in one spacious, bright, and noise-free rooms. The task duration was about 10 min. All the participants received a gift after completing all the tasks.

### Data Analysis Plan

Data were analyzed using statistical software SPSS 25.0 for Mac. Firstly, a one-way ANOVA was conducted to explore the overall development differences in the IGT among individuals aged 8–12, meanwhile, regression models with both linear and quadratic functions were conducted to explore their developmental trajectory of affective decision-making. Secondly, another two repeated-measures ANOVA were conducted to analyze how affective decision-making changed over the course of the IGT for different ages. In the first 5 × 5 ANOVA, age (8–12 years old) was set as between-participant factors and block (Block 1–5) was set as a within-participant factor. In the second 5 × 2 × 2 × 3 ANOVA, age (8–12 years old) was set as between-participant factors and gain (advantageous vs. disadvantageous choices), frequency (high vs. low frequency) and block (Block 1–2: early learning stage vs. Block 3–4: mid-learning stage vs. Block 5: final performance stage) were set as within-participant factors. Furthermore, participants were clustered into two age groups of children (8–10 years old) and early adolescents (11–12 years old), and correlations and hierarchical regressions (IQ facet scores in Step 1 and EI facet scores in Step 2) were conducted to investigate the relative contributions of IQ and EI in predicting IGT performance for children and early adolescents.

## Results

### IGT Performance

#### Overall Developmental Differences in IGT

The total net scores as an index of the IGT performance for participants aged 8–12 are shown in Table 2. The one-way ANOVA was conducted with age as the independent variable and the IGT total net score as the dependent variable. The results showed that there were no significant developmental differences regarding affective decision-making ability among the age groups, *F*(4, 182) = 1.01, *p* > 0.05. Furthermore, the simulation results of regression models were not significant (linear function: *R*^2^ = 0.010, *F*(1, 185) = 1.89, *p* > 0.05. Quadratic function: *R*^2^ = 0.012, *F*(2, 184) = 1.15, *p* > 0.05). However, it is worth mentioning that the total net score of the 12 years old IGT was the lowest, indicating that early adolescents may have a relatively poor affective decision-making ability. This finding deserved further analysis over the course of IGT.

**TABLE 2:** Net scores on total and each block in each age group (*M* ± *SD*).

Age groups (years old)	Total	Block 1	Block 2	Block 3	Block 4	Block 5
8	−2.21 ± 27.00	−0.15 ± 6.26	−2.05 ± 8.41	−0.51 ± 6.85	−0.67 ± 7.79	1.18 ± 6.88
9	−3.32 ± 24.07	−1.11 ± 6.78	−2.58 ± 8.96	−1.05 ± 7.08	0.21 ± 7.89	1.32 ± 6.09
10	−5.95 ± 20.00	−2.21 ± 4.58	−2.46 ± 6.89	−0.36 ± 5.29	−1.44 ± 6.27	0.56 ± 6.31
11	−1.94 ± 21.14	−3.22 ± 5.60	−2.89 ± 6.37	1.11 ± 7.00	1.67 ± 4.52	1.89 ± 6.23
12	−11.14 ± 20.63	−5.71 ± 6.56	−4.91 ± 7.18	−2.23 ± 5.44	−0.86 ± 6.85	2.57 ± 7.96

#### Developmental Differences Over the Course of the IGT

Two ANOVAs were conducted to explore how affective decision-making changed over the course of IGT in different ages. The means and standard deviations of total net scores of each task block across age groups are shown in Table 2.

**FIGURE 2:**
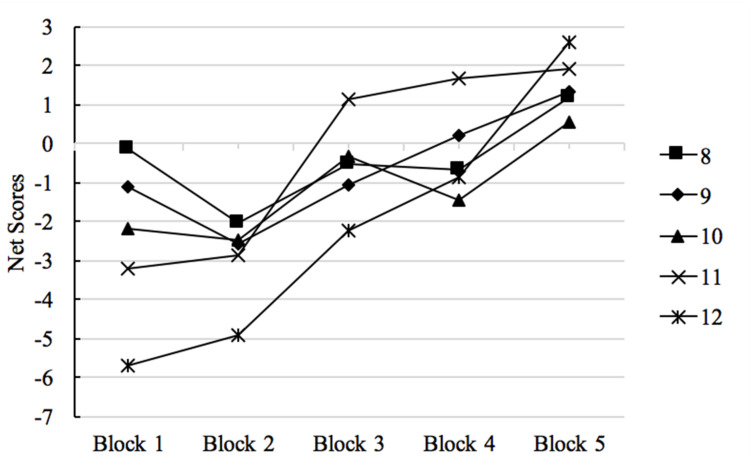
IGT performance: net advantageous choices by block across age groups.

In the first 5 × 5 ANOVA, the main effect of block was significant, *F*(4, 179) = 10.85, *p* < 0.001, η^2^ = 0.20. The interaction between block and age was significant, *F*(4, 182) = 4.54, *p* = 0.002, η^2^ = 0.09. The interaction is presented in Figure 2. *Post hoc* Bonferroni tests revealed that the 8 and 9 years olds both outperformed the 12 years olds in Block 1 (*p*s < 0.05). Moreover, for the 11 and 12 years old, their performance in Block 1 and 2 were significantly poorer than those in Block 4–5 (all *p*s < 0.05). In addition, 11 years old children performed better in Block 3 than Block 1–2, and 12 years old children performed better in Block 5 than Block 3.

**FIGURE 3:**
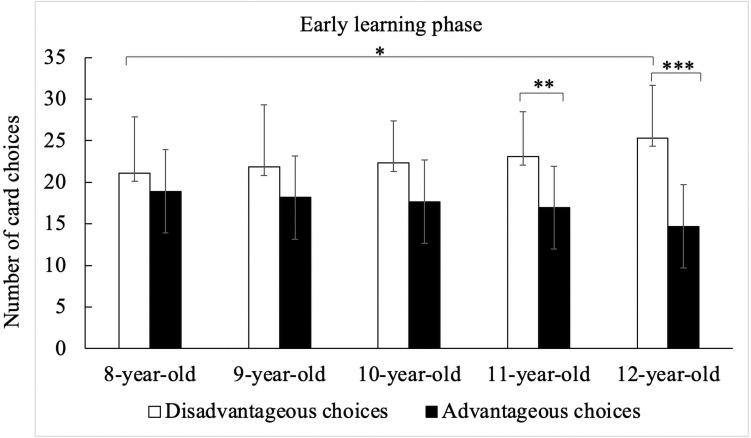
Number of card choices in early learning phase among different age groups. *p < 0.05, **p < 0.01,***p < 0.001.

**FIGURE 4:**
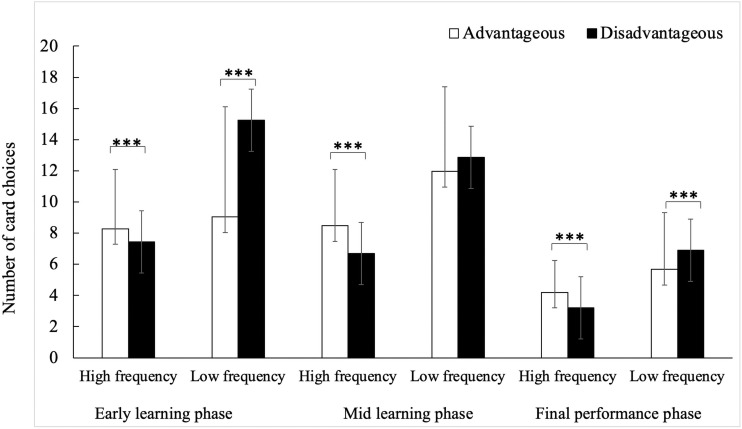
Number of card choices in different punishment frequency conditions. ***p < 0.001.

In the second 5 × 2 × 2 × 3 ANOVA, the main effects of gain [*F*(1, 182) = 8.20, *p* = 0.005, η^2^ = 0.04] and frequency [*F*(1, 182) = 189.44, *p* < 0.001, η^2^ = 0.51] were significant. Furthermore, the interactions between block and frequency [*F*(1, 182) = 18.28, *p* < 0.001, η^2^ = 0.09], between gain and frequency [*F*(1, 182) = 51.41, *p* < 0.001, η^2^ = 0.22], between gain and block [*F*(1, 182) = 28.95, *p* < 0.001, η^2^ = 0.14] were significant. There are two main discoveries in this ANOVA. The first was the significant interaction among age, gain and block, *F*(4, 182) = 1.99, *p* = 0.046, η^2^ = 0.04. As can be seen in Figure 3, *post hoc* Bonferroni tests reflected that 12 years old adolescents chose more disadvantageous cards than 8 years old children in early learning phase (Block 1–2), *p* = 0.046; 11 and 12 years old adolescents made more disadvantageous selections than advantageous ones during the early learning phase (*p* = 0.004 and *p <* 0.001, respectively), but children aged 8 and 9 both showed no such pattern. The second important result was the significant interaction among gain, frequency, and block [*F*(1, 182) = 19.31, *p* < 0.001, η^2^ = 0.10]. The *post hoc* Bonferroni tests showed that in early learning phase, mid learning phase and final performance phase, participants chose more advantageous cards than disadvantageous ones when faced with high frequency punishment conditions, but participants chose less advantageous cards than disadvantageous ones when faced with low frequency punishment conditions in early learning phase and final performance phase, *p*s < 0.001, see details in Figure 4. Participants chose less and less high punishment frequency cards during learning stages (early learning phase > mid learning phase > final performance phase), *p*s < 0.025.

### IQ, EI, and IGT in Children and Early Adolescents

Participants were clustered into two large age groups of children (8–10 years old) and early adolescents (11–12 years old). As presented in Table 3, *t*-tests were used to analyze the total and facet scores of IQ and EI in children and early adolescents. Regarding the IQ score, early adolescents had higher IQ scores than children in total IQ (*t* = –5.09, *p* < 0.001) and four facets of IQ scores (sequence reasoning: *t* = –2.83, *p* = 0.005; homogenization generalization: *t* = –4.52, *p* < 0.001; matrix reasoning: *t* = –2.69, *p* = 0.008; qualitative analysis *t* = –4.40, *p* < 0.001). Regarding the total and facet scores of EI, there was no significant difference between children and adolescents, *ps* > 0.05.

**TABLE 3:** Total scores and facets scores of IQ and EI in 8–10 years old children and 11–12 years old early adolescents.

	Children	Adolescents	*t*-tests	
	*M ± SD*	*M ± SD*	*t*	*p*
IQ total score	28.35 ± 7.53	33.01 ± 5.00	–5.09	< 0.001
IQ-sequence reasoning	8.65 ± 2.78	9.69 ± 2.22	–2.83	0.005
IQ-homogenization generalization	7.68 ± 2.93	9.35 ± 2.11	–4.52	< 0.001
IQ-matrix reasoning	8.59 ± 2.53	9.44 ± 1.79	–2.69	0.008
IQ-qualitative analysis	3.44 ± 1.64	4.54 ± 1.67	–4.40	< 0.001
EI total score	3.91 ± 0.47	3.88 ± 0.44	0.52	0.607
EI-adaptability	3.91 ± 0.66	3.97 ± 0.66	–0.65	0.516
EI-emotion expression	3.57 ± 0.83	3.37 ± 0.76	1.67	0.097
EI-emotion perception	4.01 ± 0.58	3.89 ± 0.57	1.38	0.170
EI-self-motivation	4.26 ± 0.61	4.31 ± 0.56	–0.60	0.552
EI-self-esteem	3.79 ± 0.68	3.66 ± 0.76	1.20	0.232
EI-low impulsivity	3.61 ± 0.63	3.50 ± 0.71	1.17	0.243
EI-peer relations	4.09 ± 0.61	4.15 ± 0.60	–0.63	0.529
EI-emotion regulation	3.82 ± 0.63	3.80 ± 0.59	0.14	0.889
EI-affective disposition	4.06 ± 0.88	3.88 ± 0.87	1.40	0.165

Correlation and hierarchical regression results among IQ, EI, and IGT can be seen in Tables 4–7. Tables 4, 6 were about 8–10 years old children, while Tables 5, 7 were about 10–12 years old early adolescents. Tables 5, 6 mainly showed the overview of relationships among IQ, EI, and IGT performance, in order to reveal whether IQ and EI had significant relations to IGT performance for children and early adolescents. More specific information on facets of IQ and EI in predicting children and adolescents’ IGT performance can be seen in hierarchical regressions results in Tables 6, 7.

**TABLE 4:** Correlations among IQ, EI, and the IGT performance of children aged 8–10.

	1	2	3	4	5	6	7	8	9	10	11	12	13	14	15	16	17
1. IQ total	–																
2. EI total	0.33**	–															
3. EI-Adaptability	0.34**	0.49**	–														
4. EI-Emotion expression	0.33**	0.71**	0.40**	–													
5. EI-Emotion perception	0.20*	0.69**	0.31**	0.51**	–												
6. EI-Self motivation	0.33**	0.75**	0.30**	0.42**	0.50**	–											
7. EI-Self-esteem	0.30**	0.68**	0.14	0.37**	0.42**	0.44**	–										
8. EI-Low impulsivity	0.14	0.59**	0.19*	0.33**	0.34**	0.47**	0.32**	–									
9. EI-Peer relations	0.18	0.75**	0.30**	0.46**	0.42**	0.57**	0.47**	0.26**	–								
10. EI-Emotion regulation	0.10	0.66**	0.21*	0.41**	0.38**	0.43**	0.41**	0.41**	0.39**	–							
11. EI-Affective disposition	0.15	0.79**	0.21*	0.39**	0.47**	0.54**	0.62**	0.42**	0.58**	0.51**	–						
12. IGT-Total	–0.01	–0.17	–0.08	–0.12	−0.19*	−0.20*	–0.10	0.01	−0.23*	0.10	–0.18	–					
13. IGT-Block 1	−0.23*	−0.27**	−0.23*	–0.16	−0.21*	−0.29**	−0.21*	–0.01	−0.27**	0.01	−0.25**	0.67**	–				
14. IGT-Block 2	0.01	−0.22*	−0.22*	–0.13	–0.17	−0.19*	–0.11	–0.06	−0.29**	0.08	−0.19*	0.79**	0.69**	–			
15. IGT-Block 3	0.08	–0.08	0.05	–0.03	–0.13	–0.11	–0.07	0.03	–0.12	0.07	–0.13	0.79**	0.44**	0.54**	–		
16. IGT-Block 4	0.06	–0.02	0.07	0.01	–0.15	–0.07	0.01	0.11	–0.11	0.11	–0.04	0.68**	0.22*	0.29**	0.43**	–	–
17. IGT-Block 5	0.04	–0.01	0.04	–0.10	0.01	–0.06	0.03	–0.02	0.01	0.08	–0.02	0.53**	–0.01	0.16	0.34**	0.40**	–

*p < 0.05. **p < 0.01.

**TABLE 5:** Correlations among IQ, EI, and the IGT performance of early adolescents aged 11–12.

	1	2	3	4	5	6	7	8	9	10	11	12	13	14	15	16	17
1. IQ total	–																
2. EI total	–0.12	–															
3. EI-Adaptability	0.13	0.55**	–														
4. EI-Emotion expression	–0.13	0.66**	0.29*	–													
5. EI-Emotion perception	–0.04	0.60**	0.49**	0.39**	–												
6. EI-Self motivation	–0.22	0.37**	0.17	0.08	0.08	–											
7. EI-Self-esteem	–0.09	0.80**	0.38**	0.60**	0.44**	0.27*	–										
8. EI-Low impulsivity	–0.18	0.47**	–0.04	0.15	0.10	0.26*	0.23	–									
9. EI-Peer relations	–0.05	0.83**	0.49**	0.47**	0.51**	0.15	0.67**	0.31**	–								
10. EI-Emotion regulation	–0.01	0.49**	0.09	0.28*	0.37**	0.23	0.30*	0.22	0.40**	–							
11. EI-Affective disposition	–0.15	0.75**	0.34**	0.56**	0.30**	0.07	0.60**	0.27*	0.63**	0.31**	–						
12. IGT-Total	–*0.06*	–*0.09*	*0.01*	–*0.10*	–*0.15*	*0.06*	–*0.20*	*0.23*	–*0.08*	*0.03*	–*0.10*	–					
13. IGT-Block 1	–*0.10*	*0.06*	*0.04*	*0.08*	–*0.01*	–*0.01*	–*0.11*	*0.18*	*0.07*	*0.13*	*0.04*	0.64**	–				
14. IGT-Block 2	–*0.18*	*0.12*	–*0.04*	*0.16*	–*0.06*	*0.15*	–*0.07*	*0.20*	*0.09*	0.27*	*0.10*	0.64**	0.68**	–			
15. IGT-Block 3	–*0.02*	–*0.10*	*0.02*	–*0.15*	–*0.12*	*0.06*	–*0.14*	*0.21*	–*0.12*	–*0.03*	–*0.10*	0.80**	0.35**	0.35**	–		
16. IGT-Block 4	*–0.01*	–*0.17*	–*0.01*	–*0.23*	–0.33**	*0.02*	–*0.16*	*0.12*	–*0.20*	–0.24*	–*0.17*	0.58**	0.04	0.01	0.50**	–	–
17. IGT-Block 5	*0.10*	–*0.14*	*0.01*	–*0.18*	*0.01*	–*0.02*	–*0.14*	*0.07*	–*0.10*	–*0.09*	–*0.18*	0.62**	0.04	0.04	0.43**	0.46**	–

*p < 0.05. **p < 0.01.

**TABLE 6:** Results of hierarchical regressions using IQ and EI as predictors of IGT performance in 8–10 years old children.

*DV*	Block 1		Block 2		Block 3		Block 4		Block 5		IGT total	
Independent variables	Model 1	Model 2	Model 1	Model 2	Model 1	Model 2	Model 1	Model 2	Model 1	Model 2	Model 1	Model 2
IQ-sequence reasoning	–0.06		0.07		0.01		0.07		0.07		0.05	
IQ-homogenization generalization	–0.15		–0.08		0.01		–0.03		–0.07		–0.09	
IQ-matrix reasoning	–0.19		–0.14		0.00		–0.03		0.04		–0.09	
IQ-qualitative analysis	0.15		0.21*		0.12		0.09		0.02		0.18^#^	
EI-adaptability		–0.15		–0.20		0.08		0.12		0.08		–0.02
EI-emotion expression		0.03		0.01		–0.01		0.01		−0.26*		–0.06
EI-emotion perception		–0.06		–0.06		–0.13		–0.23		0.07		–0.12
EI-self-motivation		–0.16		–0.03		–0.11		–0.10		–0.15		–0.15
EI-self-esteem		–0.05		–0.01		–0.01		0.07		0.07		0.03
EI-low impulsivity		0.13		–0.04		0.08		0.15		0.01		0.09
EI-peer relations		–0.07		–0.23		–0.05		–0.12		0.08		–0.13
EI-emotion regulation		0.22		0.31**		0.19		0.19		0.17		0.32**
EI-affective disposition		–0.21		–0.14		–0.15		–0.06		–0.08		–0.18
*F*	2.90*	2.32*	1.41	2.24*	0.45	0.76	0.32	1.00	0.21	0.59	0.93	1.72^#^
*R*^2^	0.10*	0.23*	0.05	0.22*	0.02	0.09	0.01	0.11	0.01	0.07	0.03	0.18
△*R*^2^		0.13*		0.17*		0.07		0.10		0.06		0.12^#^

*p < 0.05. **p < 0.01. ^#^Marginal significant. The coeffcients reported are standardized beta values.

**TABLE 7:** Results of hierarchical regressions using IQ and EI as predictors of IGT performance in 11–12 years old early adolescents.

DV	Block 1		Block 2		Block 3		Block 4		Block 5		IGT total	
Independent variables	Model 1	Model 2	Model 1	Model 2	Model 1	Model 2	Model 1	Model 2	Model 1	Model 2	Model 1	Model 2
IQ-Sequence reasoning	0.01		0.02		–0.07		–0.13		0.02		–0.04	
IQ-homogenization generalization	–0.05		–0.18		0.07		0.13		0.16		0.04	
IQ-matrix reasoning	0.06		–0.15		–0.16		0.04		0.06		–0.05	
IQ-qualitative analysis	–0.22		0.08		0.17		–0.08		–0.14		–0.05	
EI-adaptability		0.26		0.04		0.25		0.38*		0.12		0.30
EI-emotion expression		0.32		0.28		–0.09		–0.01		–0.04		0.14
EI-emotion perception		–0.19		–0.22		–0.13		−0.41*		0.07		–0.26
EI-self-motivation		–0.14		0.11		0.01		–0.06		–0.03		–0.03
EI-self-esteem		–0.37		−0.43*		–0.10		0.04		–0.07		–0.30
EI-low impulsivity		0.25		0.11		0.34*		0.33*		0.21		0.36*
EI-peer relations		–0.04		0.21		–0.08		–0.17		–0.09		–0.04
EI-emotion regulation		0.27		0.26*		0.02		–0.05		–0.02		0.16
EI-affective disposition		0.01		–0.02		–0.11		–0.11		–0.11		–0.11
*F*	0.80	1.25	1.19	1.47	0.68	1.00	0.53	1.67	0.80	0.52	0.14	1.08
*R*^2^	0.05	0.22	0.07	0.25	0.04	0.19	0.03	0.28^#^	0.05	0.11	0.01	0.20
△*R*^2^		0.17		0.18		0.15		0.25^#^		0.06		0.19

*p < 0.05. **p < 0.01. ^#^Marginal significant. The coeffcients reported are standardized beta values.

The correlations among IQ, EI, and the IGT performance in children and early adolescents are presented in Tables 4, 5, respectively. The results showed that for children, IQ and EI were found correlated with their IGT total net scores or net scores in Block 1 and Block 2. For early adolescents, their IGT performance was only associated with EI facets including emotion perception and emotion regulation in Block 2 and Block 4. Additionally, we found that age had no significant relations to IGT performance (net scores in total and in five blocks) within 8–10 years old children and 11–12 years old adolescent groups, *p*s > 0.05. Therefore, we haven’t treated age as a covariate in further regression analyses. To disentangle the unique contributions of IQ and EI to IGT performance for children and early adolescents, six separate hierarchical regressions (IQ scores in Step 1 and EI total scores in Step 2) were conducted for each group. As presented in Table 6, for children, IQ scores could uniquely predict their overall IGT performance and Block 2 (β = 0.18, *p* = 0.06; β = 0.21, *p* = 0.03, respectively). Even after controlling for IQ scores, EI scores remained a significant predictor of children’s IGT performance in overall IGT performance, Block 1 and Block 2 (Δ*R*^2^ = 0.12, *p* = 0.07; Δ*R*^2^ = 0.13, *p* = 0.01, and Δ*R*^2^ = 0.176, *p* = 0.01, respectively). Specially, children’s emotion regulation positively predicted their net scores in total IGT and Block 2, whereas emotion expression negatively predicted their IGT performance in Block 5.

For early adolescents, EI, rather than IQ could predict their performance in IGT, specially, self-esteem (β = –0.43, *p* = 0.02), and emotion regulation (β = 0.26, *p* = 0.04) could predict early adolescents’ IGT performance in Block 2. Moreover, lower impulsivity predicted better performance in Block 3, Block 4 and total IGT for early adolescents (β = 0.34, *p* = 0.02; β = 0.33, *p* = 0.02; β = 0.36, *p* = 0.01, respectively), and adolescents’ adaptability could predict their IGT performance in Block 4 (β = 0.38, *p* = 0.02), whereas a higher score of emotion perception could lead to less satisfactory IGT performance in Block 4 (β = –0.41, *p* = 0.01; see details in Table 7). More importantly, after controlling IQ scores, EI had a marginal unique contribution in predicting early adolescents’ IGT performance in Block 4 (Δ*R*^2^ = 0.25, *p* = 0.07).

## Discussion

The aim of the present study was to investigate the developmental differences in affective decision-making measured by the IGT during the transition from childhood into early adolescence, and the association among IQ, EI, and IGT in this period. As expected, the IGT performance of children and early adolescents (aged 8–12) progressed in a non-monotonic change way, and deficits in decision-making during early adolescence (aged 11–12) was observed, especially in their early learning phase of the IGT process. Moreover, IQ and EI played different roles in IGT performance for children and early adolescents: both IQ and EI could predict young children’s IGT performance, whereas for early adolescents, only EI contributed to their IGT performance.

Consistent with our hypothesis, the trajectory of affective decision-making ability of children and early adolescents aged 8–12, progressed in a non-monotonic change way, as the linear and the quadratic regressions failed to predict the model of development. Although we observed that the 12 years olds had the lowest mean net scores compared to the other age groups, one-way ANOVA showed no statistically developmental differences in total net scores. IGT impairment in early adolescents (Smith et al., 2012; Bernal et al., 2015) should be further explored. At the present study, through examining the IGT performance on a block-by-block basis, we could see that young children aged 8–9 surpassed the 12 years olds in the first block. Considering participants may have no sufficient learning opportunities in the first block, we combined blocks and divided overall IGT task into three phases: early leaning phase (Block 1–2), mid-learning phase (Block 3–4) and final performance (Block 5), and compared the age-related differences in different phases of IGT. More importantly, we further found that 12 years old early adolescents chose more disadvantageous cards than 8 years old children in early learning phase, and 11 and 12 years old adolescents made significantly more disadvantageous selections than advantageous ones during the learning phase, but neither children aged 8 nor 9 had such pattern. Overall, these findings suggest that early adolescents had a relatively poor affective decision-making ability as indexed by IGT performance, and the impairment demonstrated itself mainly during the early learning phase of IGT process. Previous studies concerning the imbalance model has largely reported that the heightened sensitivity of the socioemotional system could lead to the elevated risk-taking during adolescence (Blakemore, 2008; Romer, 2010; Chein et al., 2011; Quinn and Harden, 2013). In addition, it is worth mentioning that early adolescents’ average net scores for Block 5 were positive, and children and early adolescents had no significant difference in Block 5. These findings indicated that early adolescents may have a risk-taking preference only in the early learning phase, due to the fact that they were exploring the cards as much as possible. However, early adolescents performed better and better in the mid-learning and final performance phases of IGT, and their learning rate was even faster than younger children, which are similar to the prior studies (Crone and van der Molen, 2004; Van Duijvenvoorde et al., 2012). The meta-analysis by Defoe et al. (2015) showed that on the one hand, adolescents take more risks on “hot” tasks with immediate outcome feedback, which is consistent with neurodevelopmental imbalance model (i.e., Galvan et al., 2006; Pfeifer and Peake, 2012; McClure and Bickel, 2014) for investigating age differences in risky decision making. On the other hand, early adolescents took equal levels of risks as children on tasks with a sure or safe option, especially when developmental risky decision-making tasks were not confounded (i.e., mix of expected value of options and varying degree of risk). This finding could be explained by the fuzzy trace theory (FTT, Reyna and Brainerd, 2011), FTT proposed two types of mental representations occurred when people in risky decision making: verbatim representations (precise processing of words or numbers) and gist representations (bottom-line meaning processing of situation). Reyna et al. (2011) further pointed out that individual uses more gist-based decision making with age, and it allows people to better inhibit emotional impulses than interference-sensitive verbatim processing. Therefore, adolescents may take less risk-taking than children when decision making is gist-based.

Additionally, in the present study, participants chose less high punishment frequency decks with learning stages, and more advantageous decks than disadvantageous ones when faced with high frequency punishment conditions. These findings indicated that punishment frequency of decks, especially high punishment frequency ones, could help 8–12 years old children and adolescents to make advantageous choices in IGT. Hawthorne and Pierce (2015) have argued that successful IGT performance may require participants to analyze more than one dimension including the amount and frequency of gain and loss. These findings in the current study also suggested that individuals aged 8–12 may have developed a frequency awareness, meaning that they would focus on loss frequency as an IGT strategy.

In line with our hypothesis, for children aged 8–10, IQ and EI could jointly predict their IGT performance, specially, IQ scores could uniquely predict their overall IGT performance and Block 2, and EI scores remained a significant contribution in predicting children’s IGT performance in overall IGT performance, Block 1 and Block 2 when controlling for IQ scores. On the one hand, the IQ-IGT relationship in children is in accord with prior studies. For example, Li et al. (2017) found that intellectually gifted children have better IGT performance than intellectually average children. A recent study of Flouri et al. (2019) have found that children’s IQ was substantively related to the quality of decision-making measured by another gambling task—Cambridge gambling task. Furthermore, researchers proposed that affective decision-making measured by the IGT demands inductive reasoning (important aspect of IQ) from participants through the trial-and-error process of IGT (Busemeyer and Townsend, 1993). On the other hand, the SMH proposed by Damasio (1996), may well explain the EI-IGT relationship in the current study. The theory suggests that when individuals are faced with complex decision-making, somatic cells markers would guide them to make advantageous choices in uncertain situations. Research afterward confirmed that emotional processes underlie the IGT performance (Sevdalis et al., 2007; Telle et al., 2011). For early adolescents aged 11–12, surprisingly, only EI rather than IQ was found correlated to their IGT performance. According to the dual systems model, due to maturational imbalance, the socioemotional system is likely to override their cognitive control system during early adolescence (Casey et al., 2008; Steinberg, 2010; Luciana et al., 2012; Shulman et al., 2016). Hence, it is not strange to understand EI’s dominating influence in early adolescents’ affective decision-making in the current result. In addition, no age-related differences were found in EI in the present study between 8 and 10 years old children and 11–12 years old adolescents. However, some research found there were age-related differences in EI development. For example, Fili (2016) used the same EI measurement (TEIQue-CF) as the present study, and they showed that 12 years old older children had higher emotion expression scores (one facet of EI) relative to 11 years old younger children, but 10 years old younger children had higher peer-relations scores (one facet of EI) relative to 11 years old older children. Moreover, no age-related differences were found in other facts of EI (adaptability, emotion perception, and emotion regulation) in the study of Fili (2016). EI development may be an important factor which is responsible for the improvements in affective decision-making.

When it comes to facets of EI, several domains of EI predicted children and early adolescents’ IGT performance based on regression results. Interestingly, emotion regulation was found to positively affect the IGT performance in both children and early adolescents in Block 2. Emotion regulation, as a facet of EI, is described as individuals’ self-perceptions of how well they are able to control emotions (Mavroveli et al., 2008). Moreover, the SMH suggests that somatic markers play a significant role in optimizing IGT performance (Bechara et al., 1997), but negative emotions such as anxiety may impair IGT performance by hindering effective function of somatic markers (Preston et al., 2007; Miu et al., 2008). Therefore, we believe that children and early adolescents who possessed higher emotion control ability are better at dealing with difficult emotions such as anger, anxiety and stress. In this way, without interruption of these negative feelings, their somatic markers could guide optimal decision-making to the fullest. More importantly, our ANOVAs findings showed that early adolescents’ deficits mainly reflected in the early learning phase (Block 1–2) of IGT, and the relations between emotion regulation and IGT performance only existed in Block 2. Meanwhile, no age-related differences of emotion regulation were found in the present study. These findings indicated that emotion regulation may be an important factor which is responsible for the early adolescents’ deficit in early learning phase of IGT. Specially, the developmental level of emotion regulation for early adolescents is short of meeting their requirements in IGT. Therefore, the need for EI training targeted (i.e., emotion regulation) at early adolescents should be highlighted, in order to enhance their affective decision-making ability.

Furthermore, we found that the adaptability (one facet of EI) could predict successful IGT performance for early adolescents. Affective decision-making itself is a social adaptation ability that evaluates, selects and focuses on the long-term interests of the future (Cheng et al., 2013). Hence, the natural relationship between adaptability and IGT performance in early adolescents is not hard to understand. Meanwhile, the present study revealed that low impulsivity could predict early adolescents’ overall IGT performance. Mavroveli et al. (2008) hold the view that low impulsivity is one of the EI, and it refers to the ability to effectively control oneself. Some previous studies have found that the IGT performance was influenced by individual differences in impulsivity, specially, low-income children rated high impulsivity by their teachers performed less advantageously in the IGT (Burdick et al., 2013). Furthermore, higher self-reported impulsivity was significantly correlated with poorer IGT performance for college students (Sweitzer et al., 2008). Together, this evidence, combined with our findings, suggests that low impulsivity is positively associated with IGT performance. Early adolescents with lower impulsivity may be less sensitive to the easy temptation of immediate rewards from disadvantageous decks, which enables them to make wiser selections based on long-term consequences.

The SMH supports the idea that emotional processes play an essential role in the IGT, and the sensitivity to subtle emotional cues in oneself seem to correspond to the kind of EI referenced in the SMH (Damasio, 1994). However, surprisingly, the present study found that emotional expression for children and emotion perception for early adolescents would negatively predict IGT performance in Block 4 or Block 5. These findings may be a consequence of the process of cognitive awareness of IGT. During the early learning phase of the game (Block 1–2), due to the help of somatic markers (more related to emotional ability), participants were able to perform well in IGT even though they are at a pre-conscious level (Bechara et al., 1994). However, in the later mid learning phase (Block 3–4) as well as the final performance phase (Block 5), when participants have gradually formed an explicit cognitive understanding (more related to cognitive ability) of the task (i.e., knowing the deck rules), the excessive involvement of emotional expression/perception may disturb and therefore even hinder the IGT performance. These findings deserve future studies to confirm.

Some limitations of the current study should be noted. Firstly, though we ensured enough sample size of each continuous age group, we did not fully replicate linear or quadratic trajectory of IGT development as proposed by prior studies (i.e., Crone and van der Molen, 2004; Prencipe et al., 2011; Smith et al., 2012). The possible interpretation may be the high degree of variation among participants of the same age during developmental stages (Smith et al., 2012). Hence, future longitudinal study or cross-cultural work are required to identify related variables influencing affective decision-making. Secondly, Figner and Weber (2011) pointed out that impaired IGT performance in early adolescence may be due to the maturational imbalance of the overactive socioemotional system (high reward sensitivity) and the slow-developed cognitive control system, but in the present study, we discovered the correlation among IQ, EI, and IGT only by behavioral assessments in children and adolescents. Meanwhile, Defoe et al. (2015) pointed out that a part of the brain (i.e., PFC) does not explain behaviors, so further studies on this topic should include functional magnetic resonance imaging (FMRI) analysis to directly reveal the developmental neural mechanism of children and early adolescents in IGT. Finally, some results of this study are too specific, specially related to facets of IQ and EI associated with IGT progress (Blocks 1–5), such as some facets of EI among early adolescents predict their IGT performance only in Block 4. These findings make the relationship among IQ, EI, and IGT not clear-cut and lose some points of theoretical meaning to some extent. More studies are needed to confirm some specific results in the future.

In conclusion, based on the IGT performance, we found that the development of affective decision-making ability conformed to a non-monotonic change way, and there was a developmental trend of an impaired IGT performance in early adolescence, especially in the early learning phase of IGT. In addition, IQ and EI contributed differently to their IGT performance for children and early adolescents: IQ and EI jointly predict young children’s IGT performance whereas for early adolescents, only EI contributed to their IGT performance. The present study may enhance our understanding of how cognitive processing and emotional processing interact in affective decision-making from a developmental perspective. Our finding that important facets of EI (i.e., emotion regulation) positively predict IGT performance for early adolescents adds to the literature on adolescent affective decision-making ability. More importantly, it highlights the need for EI training targeted at early adolescents for guiding them safely through complicated real-world decision-making.

## Data Availability Statement

The original contributions presented in the study are included in the article/supplementary material, further inquiries can be directed to the corresponding author/s.

## Ethics Statement

The studies involving human participants were reviewed and approved by the School of Sociology and Psychology, Central University of Finance and Economics. Written informed consent to participate in this study was provided by the participants’ legal guardian/next of kin. Written informed consent was obtained from the individual(s), and minor(s)’ legal guardian/next of kin, for the publication of any potentially identifiable images or data included in this article.

## Author Contributions

DL, MWa, XZ, and JS designed the experiment. DL and XZ collected the data. DL and MWu analyzed the data and wrote the manuscript. All authors contributed to the article and approved the submitted version.

## Conflict of Interest

The authors declare that the research was conducted in the absence of any commercial or financial relationships that could be construed as a potential conflict of interest.
